# Autonomic balance and cardiovascular hemodynamic function in men with prolactinoma

**DOI:** 10.3389/fendo.2025.1701651

**Published:** 2025-10-31

**Authors:** Agnieszka Włochacz, Paweł Krzesiński, Robert Wierzbowski, Beata Uziębło-Życzkowska, Przemysław Witek, Grzegorz Zieliński, Anna Kazimierczak, Małgorzata Banak, Bartłomiej Włochacz, Grzegorz Gielerak

**Affiliations:** ^1^ Department of Cardiology and Internal Medicine, Military Institute of Medicine – National Research Institute, Warsaw, Poland; ^2^ Department of Internal Medicine, Endocrinology, and Diabetology, Medical University of Warsaw, Warsaw, Poland; ^3^ Department of Neurosurgery, Military Institute of Medicine – National Research Institute, Warsaw, Poland; ^4^ Department of Anesthesiology and Intensive Care, Legionowo Hospital, Military Institute of Medicine – National Research Institute, Warsaw, Poland

**Keywords:** autonomic balance, cardiovascular complications, heart rate variability, hemodynamic disorders, impedance cardiography, prolactinoma

## Abstract

**Introduction:**

Endocrine disorders associated with prolactinoma (PR) in men may affect the interaction between the cardiovascular and autonomic nervous system (ANS). The aim of this study was to evaluate the association of sympathetic-parasympathetic balance, assessed by heart rate variability (HRV) analysis, with cardiovascular hemodynamic function, assessed by impedance cardiography (ICG) and applanation tonometry (AT), in men with newly diagnosed PR.

**Methods:**

In this observational cohort study, 20 men with newly diagnosed PR and no significant comorbidities were included. A correlation analysis was performed on parameters assessed by ICG and AT with HRV indices assessed by 24-hour Holter ECG recordings. The ICG assessment included indicators of heart’s pumping efficiency: the acceleration index (ACI), the velocity index (VI), and the Heather index (HI). The AT assessment included aortic augmentation pressure (AP) and augmentation index (AI). Heart rate variability analysis incorporated time-domain parameters (pNN50, SDNN, SDSD, RMSSD) and frequency-domain parameters (total power (TP) and its individual frequency bands: low frequency (LF day/night) and high frequency (HF day/night), LF/HF day/night ratio). Furthermore, echocardiographic assessment was performed.

**Results:**

Men with PR demonstrated significant correlations between cardioimpedance parameters of heart’s pumping efficiency (ACI, HI, VI) with selected time- and frequency-domain parameters of HRV. Furthermore, significant correlations of central pressure values with selected time-and frequency-domain HRV parameters were found: a) higher AP corresponded with lower values of pNN50_day (R=-0.53, p=0.019), RMSSD_night (R=-0.49, p=0.033), pNN50_night (R=-0.49, p=0.034), TP_day (R=-0.53; p=0.02) and TP_ night (R=-0.67; p=0.002); b) higher AI corresponded with lower values of RMSSD_day (R=-0.46; p=0.047), SDSD_day (R=-0.47; p=0.044), pNN50_day (R=-0.53; p=0.021), RMSSD_night (R=-0.54; p=0.016), SDSD_night (R=-0.52; p=0.021), pNN50_night (R=-0.51; p=0.027), TP_day (R=-0.57; p=0.011) and TP_night (R=-0.69; p=0.002).

**Conclusions:**

In men with newly diagnosed PR, the association of poorer heart’s pumping efficiency and elevated indirect indicators of arterial stiffness with a shift away from parasympathetic influence was confirmed.

## 1 Introduction

Prolactinoma (PR) is a pituitary neuroendocrine tumor that autonomously secretes prolactin (PRL), leading to hypogonadotropic hypogonadism (1–5). Men diagnosed with PR, in contrast to women, have an increased risk of cardiovascular disease, which may be related to the delayed diagnosis, higher PRL levels, and the larger size and invasiveness of the tumor (6, 7). Furthermore, men with PR are at risk of metabolic disorders, including metabolic syndrome, glucose intolerance, hyperinsulinemia and insulin resistance, and premature atherosclerosis due to an abnormal lipid profile (8–14). The presence of hyperprolactinemia and an abnormal metabolic profile in men with PR has been demonstrated to result in vascular endothelial damage and cardiomyocyte dysfunction, which can ultimately lead to left ventricular (LV) dysfunction (15, 16). In male patients with newly diagnosed PR, subclinical left ventricular hemodynamic dysfunction was identified at an early stage of the disease (17). Despite the established proarrhythmic effect of prolactin, there is a lack of data on the prevalence of arrhythmias in patients with PR (18).

The potential anatomical substrate of arrhythmias in PR may be areas of myocardial fibrosis due to the effects of hyperprolactinemia on the myocardium. However, the subclinical effects of hyperprolactinemia in patients with newly diagnosed PR may not be detected by standard methods. Therefore, assessment of autonomic nervous system (ANS) balance, which has a significant role in regulating cardiovascular function, may be a valuable adjunct to the detailed clinical assessment of patients with PR. Despite the limitations imposed by inter-individual variability and interpretation, HRV continues to be a useful method for assessing autonomic nervous system function when compared to other available methods. The technology’s primary advantages are non-invasiveness, ease of use, widespread availability, the capacity for long-term patient monitoring, the ability to assess dynamic autonomic responses to external and internal variables, and the capacity to examine short- and long-term autonomic variability over time. These qualities render it suitable for use in various patient populations and clinical situations. An association between ANS imbalance with a shift away from parasympathetic influence and left ventricular hemodynamic dysfunction has been demonstrated in patients with another pituitary disease, acromegaly (19). An association between sympathetic-parasympathetic imbalance and increased morbidity and mortality from cardiovascular complications in patients with heart failure and coronary artery disease has also been demonstrated, but unfortunately no studies have concentrated on patients with PR (20–22). Understanding the association of heart rate variability (HRV) with the hemodynamic profile in patients with newly diagnosed PR may be useful in explaining the association of ANS balance with cardiovascular function and risk of complications, and might act as a useful new marker of cardiovascular dysfunction. A comprehensive overview of the hemodynamic status and autonomic balance can be achieved by using modern diagnostic methods such as impedance cardiography (ICG), applanation tonometry (AT) and HRV (23–25). This approach may offer significant cognitive and added value in the diagnosis of subclinical cardiovascular dysfunction in this population. Therefore, the aim of our study was to evaluate the association of HRV with hemodynamic parameters of cardiac function in patients with newly diagnosed PR without significant comorbidities.

## 2 Material and methods

### 2.1 Study population

The analysis included 20 men with newly diagnosed PR without endocrine or surgical treatment and without significant comorbidities who were enrolled in a prospective, observational study at the Military Institute of Medicine - National Research Institute. The study was performed in accordance with the Declaration of Helsinki and Good Clinical Practice (GCP), and all study participants signed an informed written consent to participate in the study. The study protocol was approved by the Bioethics Committee of the Military Institute of Medicine - National Research Institute in Warsaw (no. 76/WIM/2016).

A prolactinoma was diagnosed on the basis of the typical clinical features of hyperprolactinemia, endocrine abnormalities associated with elevated serum prolactin levels and confirmation of the presence of a pituitary tumor by pituitary magnetic resonance imaging (1, 2). Patients were enrolled in the study no later than two months following their diagnosis of a pituitary tumor.

Hormonal assessment of the pituitary gland was supplemented with adrenocorticotropic hormone (ACTH) and thyrotropic hormone (TSH) levels. In addition, testosterone levels were measured. Secondary causes of hyperprolactinemia and the effects of drugs affecting the dopaminergic system were excluded. Patients were not taking any medication affecting the function of the hypothalamic-pituitary-adrenal axis, which could affect the assessment of hemodynamic function. All patients with PR and concomitant hypertension were receiving antihypertensive treatment, either monotherapy based on vasodilators (angiotensin-converting enzyme inhibitors or angiotensin receptor blockers), or dual combination therapy with diuretics or calcium channel blockers. Their hypertension was well controlled.

The prevalence of hypertension and coexisting glucose intolerance (type 2 diabetes mellitus (2TDM), impaired fasting glucose (IFG) and impaired glucose tolerance (IGT)) was assessed.

Exclusion criteria were conditions that could adversely affect cardiovascular hemodynamics and heart rate variability parameters: chronic heart failure with moderate and reduced left ventricular ejection fraction (LVEF, <50%), acute and chronic coronary syndrome, heart rhythm other than sinus rhythm, arrhythmia, large number of artefacts or extra beats >500/day in Holter ECG assessment, history of pulmonary embolism, chronic obstructive pulmonary disease, chronic kidney disease with eGFR <60 ml/min/1.73 m2 by MDRD formula, history of stroke or transient cerebral ischemic episode, central nervous system disease, peripheral vascular disease, respiratory failure, history of endocrine or neurosurgical treatment for pituitary neuroendocrine tumor, history of head injury, lack of patient consent, patient’s condition preventing compliance with the study protocol.

### 2.2 Physical examination

A complete clinical examination was performed on all patients. The history involved a detailed clinical assessment including cardiovascular risk factors using a cardiovascular questionnaire covering: cardiovascular symptoms, comorbidities, nicotinism, family history of cardiovascular disease and current pharmacotherapy. Physical assessment included measurement of anthropometric parameters (height, weight, body mass index (BMI)), as well as measurement of heart rate (HR), systolic blood pressure (SBP) and diastolic blood pressure (DBP). An automatic device (Omron M4 Plus, Kyoto, Japan) was used to measure blood pressure (BP).

### 2.3 Impedance cardiography

Impedance cardiography is a modern, non-invasive and well-validated method of assessing the hemodynamic state of the cardiovascular system based on the phenomenon of impedance variability relative to blood flow in large arterial vessels. It allows the assessment of multiple parameters such as vascular stiffness, volemia and heart’s pumping efficiency thus indicating the mechanical efficiency of the heart in pumping blood throughout the circulatory system (26). In each patient, hemodynamic parameters were measured by ICG using a Niccomo™ device (Medis, Ilmenau, Germany). The measurements were conducted in the morning in the supine position in a quiet room in the presence of a trained nurse after a minimum of five minutes of rest. The 10-minute examination involved the continuous acquisition of ICG recordings and the automated measurement of SBP and DBP every 2 minutes using an upper arm cuff. Extensive analysis of the values of the hemodynamic parameters was performed using special software (Niccomo Software). The analysis of chest impedance variability enabled the determination of indicators that assess the pumping capacity of the heart, such as cardiac output and its index (CO, [ml/min]; CI, [ml*m 2*min 1]) and stroke volume and its index (SV, [ml]; (SI)[ml/m2]).

On the basis of impedance curve analysis and the electrocardiogram, parameters assessing myocardial contractility were measured: velocity index, depicting peak aortic blood flow (VI [1*1000 1*s 1]), acceleration index, describing peak aortic blood flow acceleration (ACI [1/100/s2]), and Heather index, characterizing the inotropic function of the heart (HI [Ohm/s2]). Hemodynamic parameters related to large arterial compliance were assessed: pulse pressure (PP), systemic vascular resistance and its index (SVR [dyn*s*cm 5]; SVRI [dyn*s*cm 5*m²]) and total arterial compliance and its index (TAC [ml/mmHg]; TACI [ml/mmHg*m2]). In addition, a parameter reflecting thoracic fluid content (TFC [1/kOhm]) was assessed. Groups at increased risk of clinical deterioration were identified based on SI values < 35 ml/m2 and TFC > 35 1*kOhm-1.

### 2.4 Applanation tonometry

Applanation tonometry is a novel method that uses pulse wave characteristics to indirectly assess central arterial pressure in the aorta. It is also used to assess markers that characterize arterial stiffness, reflecting left ventricular afterload (27). The measurements were obtained by a trained nurse in a supine position. Radial artery pressure curves were then recorded using an AT with a micromanometer on the left wrist (Millar Instruments, Houston, Texas, USA), poor-quality recordings were rejected from analysis. The radial pulse was calibrated relative to the last SBP and DBP measurement on the arm using the oscillometric module of the Niccomo device. Non-invasive applanation tonometry parameters were assessed using the SphygmoCor system (version 9.0; AtCor Medical Inc. Pty Ltd, Sydney, NSW, Australia). During the analysis, the arterial pulse waveform was processed and the correct aortic pressure curve was generated from the radial artery pulse curve. The pulse wave generated consisted of the pulse wave produced by the aorta and a reflected wave that overlapped it, causing amplification. Subsequent analysis of the parameters obtained determined the following parameters: central systolic BP (CSBP), central diastolic BP (CDBP), central pulse pressure (CPP) and augmentation pressure (AP), which is the difference between the pressure generated by the myocardium and the actual pressure present in the aorta, and the augmentation index (AI), calculated from the formula AI=AP × 100/CPP, which is the quotient of the augmentation pressure and the arterial pressure present in the aorta. AI has been demonstrated to be an indirect indicator of vascular stiffness. Elevated AI and AP was indicative of a heightened impact of reflected waves, which return more expeditiously to the heart due to augmented vascular stiffness. It has been demonstrated that higher AI is associated with an increased risk of cardiovascular disease and a higher risk of cardiovascular events, including myocardial infarction, stroke and atrial fibrillation. The value of AI is also found to be contingent on physiological conditions associated with hyperkinetic circulation. Research has demonstrated that AI levels are notably diminished in pregnant women (28–31).

### 2.5 Heart rate variability analysis

Heart rate variability analysis is a non-invasive, well-validated method to assess the influence of sympathetic and parasympathetic activity of the autonomic nervous system on heart rhythm (23). The in-hospital recording of 24-hour Holter electrocardiographic recordings was performed in each patient using LifeCard CF 3-channel digital recorders (Spacelabs Healthcare; USA). Prior to this assessment, all patients were advised to avoid physical exertion, smoking and alcohol consumption, and to rest between 10 pm and 6 am. In order to assess 24-hour HRV, the nocturnal phase was distinguished from 10 pm to 6 am and the daytime phase from 6 am to 10 pm. In patients diagnosed with sleep disorders or who have experienced a change in lifestyle, the analysis was conducted on an individual basis, with the data derived from the patients’ personal sleep diaries. Minimum, mean and maximum heart rate (HR), the occurrence of rhythm and conduction disturbances and heart rate variability (HRV) were assessed. The analysis was performed using the Pathfinder SL, HRV Advanced Option system (Spacelabs Healthcare; USA) which enabled automatic recognition of sinus rhythm and detection and marking of artifacts, premature ventricular and supraventricular beats, and segments with unintelligible signals. All markings were then manually verified by a qualified physician. Initial analysis involved the elimination of artefacts, the correction of misclassified beats, and the assessment of rhythm and conduction disturbances, as well as ST-segment changes. RR intervals between normal QRS complexes were analyzed, with intervals preceding and following extra beats excluded. For the purpose of HRV analysis, it was necessary to include only those recording segments that met the following criteria: a minimum duration of five minutes, the presence of sinus rhythm, the absence of artefacts exceeding five percent of the segment, and the absence of arrhythmias affecting the RR interval distribution. Finally, records containing artefacts (large number of extra beats >500/day) were excluded from the analysis. Frequency analysis was performed using Fast Fourier Transform after sampling using the cubic spline interpolation method and Blackman/Harris window function. From the obtained total frequency spectrum, normalized values for the LF, HF, LF/HF ratio and TP frequency ranges were used for analysis. Frequency analysis was performed for each hour of the entire day. Then, the average for the day and night and the day/night ratio were taken into account. The analysis included variables for a 24 h period (variable_24 h), during the day (variable_day) and during the night (variable_night). The utilization of this classification is predicated on the premise that the autonomic nervous system manifests circadian variation, with the typical elevation of sympathetic activity during waking hours and the heightened activity of parasympathetic tone during nocturnal periods. The 24-hour assessment provides both assessments of periods of high activity and recovery, thereby facilitating a more comprehensive evaluation of circadian variation and overall autonomic balance. The daytime assessment captures autonomic responses during daily physical activity and stress, while the nighttime assessment more accurately reflects parasympathetic activity by reducing confounding factors. The time-domain parameters analyzed were: pNN50 (proportion of NN50 divided by the total number of NNs [%]), SDNN (standard deviation of the NN interval [ms]), SDSD (standard deviation of successive differences), RMSSD (the square root of the mean of the sum of the squares of differences between adjacent NN intervals [ms]). The RMSSD and pNN50 parameters have been shown to correspond to parasympathetic ANS activity, while the SDNN parameter has been demonstrated to reflect long-term heart rate variability, with a particular focus on parasympathetic nervous system function. A frequency-domain analysis of HRV was performed using a fast Fourier transform (FFT), which included total power (TP, variance of all NN interval) and power in the low frequency range (LF day/night, low frequency component: 0.05-0.15 Hz), high-frequency power (HF day/night, high-frequency component: 0.15-0.35 Hz) and LF/HF day/night ratio). The HF parameter was found to correspond to parasympathetic activity, the LF parameter to both sympathetic and parasympathetic activity, and the relationship between LF and HF reflected the balance between the sympathetic and parasympathetic systems.

### 2.6 Echocardiography

All patients underwent two-dimensional (2D) echocardiography using a 2.5 MHz transducer (VIVID E95, GE Medical System, Wauwatosa, WI, USA) in standard parasternal, apical and subcostal projections, in accordance with the American Echocardiographic Society guidelines. In the parasternal long-axis view, left ventricular end-diastolic diameter (LVEDd), right ventricular end-diastolic diameter (RVEDd), interventricular septal thickness and left atrial (LA) dimension were measured. The width of the ascending aorta was measured, and the morphology and function of the heart valves and pericardium were assessed. Using a two-dimensional image of the left ventricle during systole and diastole in the apical four-chamber and two-chamber views, left ventricular ejection fraction was measured using the biplane Simpson method. The assessment of left ventricular diastolic dysfunction was conducted in accordance with current guidelines (32, 33).

### 2.7 Statistics

Statistical analysis was performed using MS Office software and Statistica 12.0 (StatSoft Inc., Tulsa, USA). For continuous variables, results were expressed as mean ± standard deviation (SD), median and interquartile range, while for categorical (qualitative) variables, results were expressed as absolute (n) and relative (%) values. The distribution of continuous variables was assessed visually and the Shapiro-Wilk test was used. Correlations between variables were assessed using Spearman correlations. A p < 0.05 was considered statistically significant.

## 3 Results

### 3.1 Baseline characteristics

The characteristics of the study group are presented in **Tables 1** and **2**.

**Table 1:** Baseline characteristics of patients with prolactinoma.

Variable	Mean ± SD (median; interquartile range) or n (%)
Age [years]	43 ± 13 (42; 31 - 52)
Male	20 (100)
BMI [kg/m^2^]	32 ± 7 (30; 28 - 35)
BMI 18.5 - 24.9 kg/m^2^	2 (10)
BMI 25 - 29.9 kg/m^2^	8 (40)
BMI ≥ 30 kg/m^2^	10 (50)
HR [bpm]	68 ± 10 (69; 61 - 74)
SBP [mmHg]	117 ± 17 (119; 102 - 130)
SBP ≥ 140mmHg	2 (10)
DBP [mmHg]	76 ± 10 (77; 66 - 82)
DBP ≥ 90mmHg	2 (10)
AH	8 (40)
DM	3 (15)
creatinine [mg/dl]	0.80
LVEF [%]	64 ± 3 (64; 63 - 65)
testosterone	1.88 ± 1.32 (1.58; 1.19 - 2.74)
prolactin	844.0 ± 1205 (202.4; 103.3 - 1074)
TSH	2.51 ± 4.69 (1.43; 0.79 - 2.17)
fT3	4.66 ± 0.41 (4.85; 4.29 - 4.94)
fT4	13.60 ± 2.61 (14.26; 11.09 - 15.37)

AH, arterial hypertension; BMI, body mass index; DBP, diastolic blood pressure; DM, diabetes mellitus; fT3, tri-iodothyronine; fT4, thyroxine; HR, heart rate; RAAB, renin-angiotensin-aldosterone blockers; SBP, systolic blood pressure; SD, standard deviation; LVEF, left ventricle ejection fraction; TSH, thyrotropic hormone levels.

**Table 2:** Hemodynamic parameters assessed by impedance cardiography and applanation tonometry in patients with prolactinoma.

Variable	Mean ± SD (median; interquartile range) or n (%)
Impedance cardiography	
SI [ml/m^2^]	46,6 ± 10,9 (44,5; 39,5-53)
CI [ml*m^-2^*min^-1^]	3,06 ± 0,55 (3; 2,65-3,35)
SVRI [dyn*s*cm^-5^*m²]	2127 ± 402,1 (2091; 1810-2378)
TAC [ml*mmHg^-1^]	2,58 ± 0,87 (2,4; 1,9-3,3)
VI [1*1000^-1^*s^-1^]	42,7 ± 12,3 (42,5; 35,5-49,5)
ACI [1/100/s^2^]	67,2 ± 22,4 (69,5; 53-81)
HI [Ohm/s^2^]	10,4 ± 2,98 (10; 8,3-12,1)
TFC [1/kOhm]	30,2 ± 4,36 (30,8; 27-33)
Applanation tonometry	
Aortic Alx (AP/PP) [%]=AI	14,4 ± 14,3 (11; 4-24)
Aortic Augmentation (AP)[mmHg]	4,32 ± 4,93 (3; 1-7)

ACI, acceleration index; AI, augmentation index; AP, augmentation pressure; CI, cardiac index; HI, Heather index; SI, stroke index; SVRI, systemic vascular resistance index; TAC, total artery compliance; TFC, thoracic fluid content; VI, velocity index.

The study group consisted of young and middle-aged men (43 ± 13 years) with a newly diagnosed prolactin-secreting pituitary neuroendocrine tumor who had not received prior endocrine or neurosurgical treatment.

Hypertension was previously diagnosed in eight patients (40%), but was effectively managed with pharmaceutical interventions. The mean blood pressure (BP) values recorded were 116/76 mmHg, with 90% of patients presenting with BP < 140/90 mmHg. The mean heart rate was 68/min. Furthermore, 90% of patients had a body mass index > 25 kg/m2, indicating weight abnormalities, with a mean BMI of 32 ± 7 kg/m2.

The prevalence of obesity was observed in 10 patients (50%), while 8 patients (40%) had a BMI classification of overweight. Type 2 diabetes was confirmed in 3 of 20 patients (15%), and prediabetic status (IFG or IGT) was present in 4 patients (20%). In the group of patients with diabetes, two patients were treated with metformin, while one patient was treated with metformin and insulin. Nineteen of the 20 patients had preserved anterior pituitary gland function. One patient was diagnosed with thyrotropin deficiency, but this was well controlled with a stable dose of L-thyroxine.

Assessment of left ventricular ejection fraction by standard echocardiography showed normal values in all patients, with a mean score of 63.7%. A detailed characterization of the study group was previously published by the authors (17).

### 3.2 Correlation between HRV variables and impedance cardiography parameters

Time-domain analysis of HRV showed significant correlations of age with lower values of RMSSD_day (R=-0.61; p=0.004), SDSD_day (R=-0.63; p=0.003), pNN50_day (R=-0.48, p=0.032) and RMSSD_night (R=-0.46; p=0.039), SDSD_night (R=-0.46; p=0.46), pNN50_night (R=-0.47; p=0.034).

Furthermore, significant correlations of selected parameters of heart’s pumping efficiency with selected HRV time-domain values were demonstrated: lower VI corresponded with lower SDNN day (R=0.46; p=0.042), lower ACI with lower SDNN day (R=0.50; p=0.023), lower HI with lower RMSSD_day (R=0.50; p=0.031) and lower pNN50_day (R=0.49; p=0.033).

HRV frequency-domain analysis showed only significant correlations of age with lower TP_day (R=-0.53; p=0.016) and TP_night (R=-0.67; p=0.002) and significant correlations of heart’s pumping efficiency (lower HI) with HRV frequency-domain index (lower TP_day (R=0.47; p=0.044)).

There were no statistically significant correlations of other HRV frequency-domain parameters (LF day/night, HF day/night and LF/HF day/night ratio) with the cardiovascular hemodynamic profile assessed by ICG (SI, VI, ACI, HI).

Detailed results are presented in **Tables 3** and **4**.

**Table 3:** Correlations between time-domain parameters of heart rate variability and impedance cardiography parameters and applanation tonometry parameters in patients with prolactinoma.

Correlations: R (p)								
	SDNN day	RMSSD_day	SDSD_day	pNN50_day	SDNN_night	RMSSD_night	SDSD_night	pNN50_night
Demographic and clinical data								
Age	-0.42 (0.068)	-0.61 (0.004)	-0.63 (0.003)	-0.67 (0.001)	-0.35 (0.127)	-0.48 (0.032)	-0.46 (0.039)	-0.47 (0.034)
BMI	-0.24 (0.309)	0.18 (0.444)	0.30 (0.193)	0.06 (0.806)	0.10 (0.673)	0.48 (0.032)	0.54 (0.014)	0.37 (0.108)
Impedance cardiography								
HR [bpm]	-0.41 (0.070)	-0.33 (0.155)	-0.29 (0.223)	-0.31 (0.183)	-0.16 (0.502)	-0.27 (0.249)	-0.35 (0.132)	-0.20 (0.402)
SBP [mm Hg]	-0.40 (0.081)	-0.10 (0.686)	-0.07 (0.756)	-0.18 (0.452)	0.03 (0.904)	0.08 (0.732)	0.11 (0.650)	0.06 (0.800)
DBP [mm Hg]	-0.44 (0.055)	-0.40 (0.084)	-0.37 (0.105)	-0.39 (0.086)	-0.02 (0.947)	-0.13 (0.595)	-0.15 (0.522)	-0.08 (0.747)
PP [mm Hg]	-0.33 (0.151)	0.11 (0.631)	0.17 (0.487)	0.03 (0.914)	0.06 (0.818)	0.21 (0.375)	0.25 (0.278)	0.15 (0.536)
SI [mL/m^2^]	0.22 (0.357)	0.27 (0.244)	0.27 (0.247)	0.30 (0.200)	0.10 (0.670)	0.23 (0.335)	0.31 (0.187)	0.17 (0.476)
CI [mL*m^-2^*min^-1^]	-0.09 (0.698)	0.13 (0.591)	0.17 (0.475)	0.13 (0.581)	0.00 (0.995)	0.14 (0.549)	0.20 (0.394)	0.09 (0.702)
VI [1*1000^-1^*s^-1^]	0.46 (0.042)	0.36 (0.124)	0.25 (0.289)	0.44 (0.051)	0.27 (0.249)	0.17 (0.461)	0.19 (0.423)	0.20 (0.405)
ACI [1/100/s^2^]	0.50 (0.023)	0.30 (0.194)	0.16 (0.503)	0.41 (0.076)	0.29 (0.219)	0.16 (0.501)	0.16 (0.497)	0.20 (0.395)
HI [Ohm/s^2^]	0.38 (0.105)	0.50 (0.031)	0.43 (0.068)	0.49 (0.033)	0.36 (0.134)	0.22 (0.370)	0.27 (0.266)	0.19 (0.441)
SVRI [dyn*s*cm^-5^*m²]	-0.22 (0,359)	-0.33 (0.158)	-0.34 (0.145)	-0.39 (0.090)	-0.03 (0.900)	-0.14 (0.561)	-0.18 (0.446)	-0.10 (0.677)
TACI [mL/mmHg*m2]	0.33 (0.169)	0.10 (0.679)	0.07 (0.787)	0.12 (0.638)	-0.03 (0.891)	0.00 (0.991)	0.04 (0.874)	-0.04 (0.874)
TFC [1/kOhm]	0.00 (0.987)	0.04 (0.872)	-0.05 (0.818)	0.05 (0.827)	0.14 (0.556)	0.07 (0.759)	0.02 (0.922)	0.11 (0.649)
Applanation tonometry								
CSBP [mmHg]	-0.47 (0.040)	-0.28 (0.252)	-0.24 (0.314)	-0.31 (0.198)	-0.09 (0.713)	-0.08 (0.734)	-0.05 (0.833)	-0.08 (0.742)
CDBP [mmHg]	-0.43 (0.067)	-0.40 (0.088)	-0.39 (0.103)	-0.37 (0.117)	-0.02 (0.943)	-0.13 (0.605)	-0.16 (0.522)	-0.06 (0.822)
CPP [mmHg]	-0.38 (0.105)	-0.08 (0.754)	0.00 (0.991)	-0.16 (0.503)	-0.14 (0.575)	-0.03 (0.898)	0.06 (0.811)	-0.09 (0.704)
AP	-0.33 (0.162)	-0.44 (0.059)	-0.42 (0.072)	-0.53 (0.019)	-0.35 (0.137)	-0.49 (0.033)	-0.44 (0.057)	-0.49 (0.034)
AI	-0.27 (0.265)	-0.46 (0.047)	-0.47 (0.044)	-0.53 (0.021)	-0.35 (0.140)	-0.54 (0.016)	-0.52 (0.021)	-0.51 (0.027)

ACI, acceleration index; AI, augmentation index; AP, augmentation pressure; CDBP, central diastolic blood pressure; CI, cardiac index; CPP, central pulse pressure; CSBP, central systolic blood pressure; DBP, diastolic blood pressure; HI, Heather index; HR, heart rate; MBP, mean blood pressure; PP, pulse pressure; SBP, systolic blood pressure; SI, stroke index; SVRI, systemic vascular resistance index; TAC, total artery compliance; TFC, thoracic fluid content; VI, velocity index; TP, total power; LF, low frequency; HF, high frequency, LF/HF, the ratio of low-frequency oscillations to high-frequency oscillations.

**Table 4:** Correlations between frequency-domain parameters of heart rate variability and impedance cardiography parameters and applanation tonometry parameters in patients with prolactinoma.

Correlations: R (p)								
	LF_day	LF_night	HF_day	HF_night	LF/HF_day	LF/HF_night	TP_day	TP_night
Demographic and clinical data								
Age	0.14 (0.547)	0.05 (0.846)	-0.28 (0.234)	-0.42 (0.065)	0.20 (0.391)	0.27 (0.254)	-0.53 (0.016)	-0.67 (0.002)
BMI	-0.25 (0.289)	-0.45 (0.045)	0.17 (0.478)	0.41 (0.073)	-0.21 (0.373)	-0.32 (0.168)	0.16 (0.510)	0.00 (0.994)
Impedance cardiography								
HR [bpm]	0.05 (0.820)	0.04 (0.876)	-0.14 (0.544)	-0.15 (0.529)	0.03 (0.889)	0.30 (0.204)	-0.26 (0.272)	-0.13 (0.607)
SBP [mm Hg]	0.10 (0.689)	-0.15 (0.534)	-0.23 (0.332)	0.10 (0.666)	0.14 (0.559)	-0.01 (0.955)	-0.10 (0.671)	-0.27 (0.269)
DBP [mm Hg]	0.36 (0.122)	0.14 (0.559)	-0.45 (0.048)	-0.17 (0.481)	0.37 (0.105)	0.34 (0.143)	-0.35 (0.131)	-0.23 (0.343)
PP [mm Hg]	-0.22 (0.351)	-0.43 (0.057)	0.13 (0.581)	0.32 (0.171)	-0.21 (0.386)	-0.29 (0.219)	0.08 (0.726)	-0.14 (0.575)
SI [mL/m^2^]	-0.04 (0.859)	-0.01 (0.971)	0.03 (0.902)	-0.07 (0.784)	0.00 (0.995)	-0.08 (0.750)	0.23 (0.329)	0.11 (0.644)
CI [mL*m^-2^*min^-1^]	-0.07 (0.767)	-0.09 (0.692)	-0.02 (0.935)	-0.11 (0.651)	-0.05 (0.848)	0.04 (0.859)	0.10 (0.677)	-0.02 (0.946)
VI [1*1000^-1^*s^-1^]	0.03 (0.895)	0.07 (0.762)	0.09 (0.717)	-0.19 (0.423)	-0.05 (0.820)	0.00 (0.985)	0.37 (0.113)	0.29 (0.222)
ACI [1/100/s^2^]	0.00 (0.997)	0.15 (0.533)	0.14 (0.558)	-0.15 (0.541)	-0.11 (0.656)	0.00 (0.995)	0.35 (0.133)	0.34 (0.148)
HI [Ohm/s^2^]	-0.04 (0.867)	0.16 (0.526)	0.15 (0.551)	-0.26 (0.279)	-0.11 (0.657)	0.19 (0.437)	0.47 (0.044)	0.39 (0.113)
SVRI [dyn*s*cm^-5^*m²]	0.21 (0.373)	0.04 (0.870)	-0.18 (0.435)	0.14 (0.548)	0.21 (0.376)	0.00 (1.000)	-0.31 (0.191)	-0.20 (0.408)
TACI [mL/mmHg*m2]	0.03 (0.914)	0.23 (0.344)	0.09 (0.712)	-0.20 (0.423)	0.03 (0.894)	0.11 (0.656)	0.06 (0.795)	0.07 (0.782)
TFC [1/kOhm]	0.01 (0.952)	-0.17 (0.478)	-0.02 (0.927)	0.00 (0.985)	-0.08 (0.733)	-0.16 (0.506)	0.14 (0.549)	-0.17 (0.490)
Applanation tonometry								
CSBP [mmHg]	0.12 (0.632)	-0.11 (0.657)	-0.27 (0.263)	0.00 (0.989)	0.17 (0.477)	0.06 (0.817)	-0.29 (0.220)	-0.37 (0.135)
CDBP [mmHg]	0.41 (0.084)	0.17 (0.495)	-0.50 (0.027)	-0.19 (0.438)	0.43 (0.068)	0.37 (0.121)	-0.36 (0.124)	-0.23 (0.361)
CPP [mmHg]	-0.28 (0.241)	-0.31 (0.197)	0.17 (0.499)	0.20 (0.414)	-0.20 (0.402)	-0.21 (0.396)	-0.18 (0.467)	-0.37 (0.129)
AP	-0.24 (0.328)	-0.19 (0.427)	0.18 (0.457)	0.03 (0.908)	-0.20 (0.401)	-0.08 (0.757)	-0.53 (0.02)	-0.67 (0.002)
AI	-0.18 (0.453)	-0.10 (0.673)	0.11 (0.646)	-0.01 (0.980)	-0.14 (0.575)	0.00 (0.989)	-0.57 (0.011)	-0.69 (0.002)

ACI, acceleration index; AI, augmentation index; AP, augmentation pressure; CDBP, central diastolic blood pressure; CI, cardiac index; CPP, central pulse pressure; CSBP, central systolic blood pressure; DBP, diastolic blood pressure; HI, Heather index; HR, heart rate; MBP, mean blood pressure; PP, pulse pressure; SBP, systolic blood pressure; SI, stroke index; SVRI, systemic vascular resistance index; TAC, total artery compliance; TFC, thoracic fluid content; VI, velocity index; TP, total power; LF, low frequency; HF, high frequency, LF/HF, the ratio of low-frequency oscillations to high-frequency oscillations.

### 3.3 Correlation between HRV variables and parameters of applanation tonometry

Significant correlations of central pressure values with selected time-domain HRV parameters were found: higher AP corresponded with lower values of pNN50_day (R=-0.53, p=0.019), RMSSD_night (R=-0.49, p=0.033), pNN50_night (R=-0.49, p=0.034), higher AI corresponded with lower values of RMSSD_day (R=-0.46; p=0.047), SDSD_day (R=-0.47; p=0.044), pNN50_day (R=-0.53; p=0.021) and lower values of RMSSD_night (R=-0.54; p=0.016), SDSD_night (R=-0.52; p=0.021), pNN50_night (R=-0.51; p=0.027).

Significant correlations of central pressure parameters with selected HRV frequency-domain parameters were observed: higher AP corresponded with lower values of TP_day (R=-0.53; p=0.02) and TP_ night (R=-0.67; p=0.002), higher AI corresponded with lower values of TP_day (R=-0.57; p=0.011) and TP_night (R=-0.69; p=0.002).

Detailed results are presented in **Tables 3** and **4**.

## 4 Discussion

In the present study, an investigation was conducted on a group of young and middle-aged male patients who had recently been diagnosed with prolactinoma. We proved the existence of some correlations between impaired heart’s pumping efficiency and increased amplification index with an imbalance of the autonomic nervous system. We revealed significant correlations of age and cardioimpedance determinants of pumping cardiac function with time- and frequency-domain parameters of HRV. Furthermore, we demonstrated significant correlations of HRV parameters with central arterial pressure parameters assessed by applanation tonometry.

It is suggested that long-term exposure to elevated prolactin levels, associated with delayed detection of pituitary tumors, may play an important role in this phenomenon. Prolactin is a hormone that acts in a multidirectional endocrine and paracrine-autocrine manner, causing immunomodulation, neurotransmission and regulation of water-electrolyte balance in addition to stimulating lactation (34). Prolactin exerts both direct positive inotropic and chronotropic effects on mammalian myocardium and indirectly via signaling pathways using cAMP as a secondary transmitter (35). It may also exert additional effects on cardiac repolarization through chronic changes in the transcription and expression of genes encoding ion channels and calcium processing proteins (18, 36). In animal studies, PRL levels have been demonstrated to be elevated in myocardial infarction and associated with increased cardiovascular smooth muscle reactivity to norepinephrine and angiotensin (37). Another study confirmed that PRL prolongs cardiac repolarization at the cellular level in LQT2 syndrome, affecting ion channels and calcium and ryanodine receptors, which may contribute to an increased risk of arrhythmia (18).

Little information is available on the cardiovascular consequences of hyperprolactinemia in humans. Further research is required to elucidate the effects of chronic hyperprolactinemia on cardiac structure, function and repolarization abnormalities. It has been hypothesized that elevated prolactin levels may be a critical factor in the pathophysiology of peripartum cardiomyopathy (38). Recent studies have demonstrated that hyperprolactinemia can induce inflammation and vascular endothelial damage, contributing to a proatherogenic environment and systemic inflammatory response (8–14). Furthermore, by increasing the expression of receptors for prolactin in cardiac myocytes and enhancing myofilament sensitivity to calcium in cardiomyocytes, it can cause cardiomyocyte dysfunction, ultimately leading to left ventricular dysfunction (39).

The few available data suggest that hyperprolactinemia in men may be associated with metabolic co-morbidities, premature atherosclerosis, vascular endothelial dysfunction and impaired vasoconstriction, with a consequent role in the development of cardiovascular disorders, although the etiology of this gender-specific finding remains to be clarified (6, 40, 41). It is probably related to the adverse effects of increased PRL levels on the function of the hypothalamic-pituitary-gonadal axis, which in men is associated with testosterone deficiency and metabolic complications of hypogonadism. Such abnormalities have not been reported in women with prolactinoma, possibly due to the physiological fluctuations of PRL associated with the menstrual cycle. In addition, subclinical cardiac dysfunction, characterized by impaired left ventricular systolic and diastolic function, has been observed in untreated patients with hyperprolactinemia (15, 42). The underlying cause of arrhythmia in patients with prolactinoma appears to be myocardial fibrosis resulting from the effects of hyperprolactinemia. Unfortunately, the subclinical effects of hyperprolactinemia in patients with newly diagnosed prolactinoma may not be detectable by standard methods, so new non-invasive markers of prognostic significance should be searched for to reflect subclinical cardiac dysfunction in this group of patients.

In our previous publication, which employed ICG, we identified an unfavorable cardiovascular hemodynamic profile in a group of men in early-stage PR. This profile was characterized by higher chest fluid content and lower stroke volume index, which could be due to early cardiovascular dysfunction and abnormalities in fluid distribution (17). The present study aimed to assess the relationship between heart rate variability and cardiovascular hemodynamic profile in young and middle-aged men with PR. Heart rate variability has been identified as a sensitive marker of cardiovascular dysfunction associated with abnormal autonomic balance (20–22). The available literature is lacking in data indicating a greater prognostic value of HRV analysis compared to traditional markers of cardiovascular mortality risk in patients with PR. The full potential of this diagnostic method has yet to be realized in the evaluation of patients diagnosed with PR. It is evident that HRV has the capacity to furnish researchers with invaluable data on the interaction between hypothalamic-pituitary axis dysfunction, autonomic dysregulation, and systemic stress in patients with PR. It has been hypothesized that HRV may also be useful in the evaluation of patients with newly diagnosed PR, as a means of detecting subclinical circulatory system disturbances in the early stages of the disease and monitoring treatment. Furthermore, it has the potential to serve as a valuable adjunct to established markers and research methods, such as echocardiography, thereby enhancing the diagnostic efficacy of HRV. It is noteworthy that the present study included men in the early stages of PR, without clinically significant cardiovascular dysfunction. All patients had normal left ventricular ejection fraction, as assessed by echocardiography. The comprehensive cardiovascular assessment we applied using HRV, ICG and AT as markers of cardiovascular dysfunction is the first attempt to use these methods simultaneously in this group of patients.

In the present study, a significant correlation was demonstrated between age and selected HRV time- and frequency-domain parameters. Despite the heterogeneity of the research methodologies employed, extant data from the literature indicate the potential role of age as an independent confounding factor influencing ANS activity. German researchers observed a significant decline in 24-hour HRV parameters (p < 0.001) and a strong negative correlation (p < 0.001) with age (43). Similar correlations of HRV parameters with age were also demonstrated by Fukusaki et al. (44). Abhishekh et al. demonstrated a correlation between age and spectral analysis parameters: negative with HF (p=0.02) and positive with the LF/HF ratio (p < 0.01), suggesting a disturbance of autonomic balance with a shift toward lower parasympathetic nervous system activity with age (45). Almeida-Santos MA et al. demonstrated an age-related decline in HRV parameters, with lower values in women than in men, reaching a nadir in the seventh decade of life. The lowest values for all variables were obtained in the group of patients with 2TDM, and no effect of both hypertension and dyslipidemia was found (46). Our study population was relatively young (31–52 years); therefore, age as a modulating variable probably plays a lesser role in this respect compared to older populations. This finding may indicate an important impact of prolonged tissue exposure to excessive PRL concentrations, associated with delayed diagnosis, which increases the risk of metabolic disorders and premature atherosclerosis (6, 8–14, 40, 41).

The potential role of hypertension and T2DM as independent confounding factors influencing ANS activity should also be considered, particularly in cases of long-term and untreated disease. These represent highly prevalent comorbidities associated with PR. De Andrade et al. found that patients with hypertension aged over 65 years had lower HRV parameters than healthy individuals (47). Mori et al. measured HRV indices by recording the R-R interval over a period of five minutes in 3,458 individuals. In men, HRV indices were not associated with SBP levels. Multivariate analysis revealed associations between SDNN, rMSSD, LF, HF and the LH/HF ratio with DBP levels in both sexes. HRV-BP correlations were not statistically significant in individuals receiving antihypertensive treatment (48). In the ARIC study, higher BP was associated with significantly lower HRV, and the correlations were stronger for DBP; moreover, the differences were greater in individuals not receiving antihypertensive treatment. Over a 9-year period, no difference in the rate of change in HRV was observed between individuals with and without hypertension (49). The Framingham study found an inverse relationship between a decrease in LF and an increase in SBP and DBP in both sexes (50).

A meta-analysis showed that T2DM was associated with decreased HRV compared to healthy controls, suggesting ANS dysfunction (51). Hyperglycemia negatively impacts ANS function, and this effect is more pronounced in patients with long-standing T2DM. The most significant decrease in HRV associated with T2DM was observed within 5 to 10 years of disease progression (52). In our study, only a small percentage of participants had T2DM and hypertension, and these conditions were well controlled (mean BP was 117/76 mmHg). By carefully selecting the study group to exclude patients with significant cardiovascular disease, we attempted to eliminate the potential influence of these factors on the studied parameters. Therefore, the methodology adopted in our study enables us to relatively unambiguously assess the effect of PR on the combined function of the ANS and cardiovascular systems.

Studies have also revealed ANS imbalances in patients with acromegaly, another pituitary disease (53–56). Multivariate linear regression analysis showed that acromegaly is significantly associated with cardiac autonomic dysfunction independently of T2DM (55). In our previous study of patients with newly diagnosed acromegaly, we confirmed significant correlations between HRV indices and the hemodynamic profile assessed by ICG (19). A relationship between HRV and ICG indices was also confirmed in patients with hypertension, but unfortunately, data in the literature are scarce (57). In contrast to our earlier observations in patients with active acromegaly (19), the hemodynamic profile exhibited a less pronounced association with HRV in men with prolactinoma. However, an association was identified between parameters of impaired heart’s pumping efficiency (VI, ACI, HI) and a shift away from parasympathetic influence. This may indicate early subclinical cardiovascular dysfunction in men with PR. This analysis, based on observational data, does not allow for an unambiguous determination of cause-and-effect relationships in this endocrinopathy. The elucidation of the mechanisms underpinning interdependence has the potential to inaugurate a novel research direction. It is evident that prolactinoma has the potential to induce imbalances within the autonomic nervous system, which may consequently result in the occurrence of cardiovascular complications. Conversely, alterations in the cardiovascular system in pituitary adenoma may also precipitate autonomic imbalances. The potential adverse hemodynamic consequences of autonomic nervous system dysfunction may lead to the development and progression of cardiovascular complications in patients with PR.

Of particular interest were the results of the correlation of HRV analysis parameters with applanation tonometry parameters. A significant correlation was demonstrated between the amplification index and the amplification pressure and HRV. In men with newly diagnosed PR, we confirmed the association of these parameters with autonomic imbalance with a shift away from parasympathetic influence. These findings suggest that early in PR, before the development of advanced complications, there is cardiovascular hemodynamic dysfunction and impaired ANS balance. However, determine cause-and-effect relationships in this endocrinopathy is a very difficult task.

Our results are consistent with the observations of other authors. Data from the literature indicate that ANS dysfunction manifested by increased sympathetic nervous system activity plays an important role in the pathophysiology of heart failure, coronary artery disease and hypertension, leading to the development of cardiovascular complications (20–22, 58). Long-term unsustainable sympathetic hyperactivity causes distant hemodynamic effects, leading to cardiac dysfunction and increased risk of sudden cardiac death and ventricular arrhythmias, becoming both a substrate and a trigger for cardiovascular morbidity and mortality (58, 59). Studies using HRV in patients with cardiovascular disease and endocrine disorders have demonstrated increased sympathetic nervous system activity already in the early stages of these conditions. In the later stages of these diseases with coexisting organ complications, a greater degree of ANS dysfunction was observed (19–22, 58–61).

### 4.1 Clinical implications

The novelty of our approach lies in the use of non-invasive cardiovascular hemodynamic assessment tools (HRV, ICG, AT) to detect subclinical PR-related abnormalities. Our observations on the effect of PRL on cardiovascular function have shed new light on the association of hyperprolactinemia with an impaired hemodynamic profile, which may result in the early development of cardiovascular complications. The association found between HRV and the hemodynamic profile assessed by ICG and AT suggests to new targets for therapy, including methods that modulate ANS function.

The establishment of an early diagnosis of cardiovascular complications, even in the absence of clinical symptoms, is a crucial and invaluable method of identifying patients who require closer cardiovascular monitoring. It is noteworthy that the non-invasive cardiovascular assessment methods utilized in this study can be readily implemented in both inpatient and outpatient care settings. However, it should be acknowledged that additional long-term monitoring of patients with PR is necessary to fully evaluate the long-term consequences of subclinical dysfunction heart’s pumping efficiency. The limitations of the HRV method are attributable to its dependence on internal and external factors, including age, gender, emotional state, circadian rhythm, consumption of stimulants and medications, individual variability, as well as nonspecificity of measurements and the lack of standardization of measurement methods. Notwithstanding this limitation, HRV remains a valuable tool in the assessment of autonomic nervous system status, albeit one that should be employed within a clinical context, with consideration for individual variability. The integration of HRV with other, more established markers and diagnostic tests has the potential to enhance its role as a diagnostic tool in the future.

### 4.2 Study limitations

The main limitation of our study was the relatively small group size due to the low incidence of PR among men. We included patients with PR without clinically significant cardiovascular dysfunction, excluding patients with serious comorbidities at recruitment. This ensured homogeneity of the study group and reduced the disruptive effect of comorbidities on ANS function and cardiovascular hemodynamics. In interpreting the results, the potential impact of coexisting hypertension (despite its good control) and the hypotensive therapy used should be taken into account. Our study included mainly young and middle-aged patients with PR, so our conclusions should not be extrapolated to the general population. Also, the effect of gender (women were not included in this study) on hemodynamic abnormalities in hormonally active PRL-secreting pituitary tumors requires further investigation. A limitation of the present study is the absence of a control group. We acknowledge the potential role of hypertension, type 2 diabetes, obesity, and age as confounding factors that influence sympathetic activity, shift away from parasympathetic influence and arterial stiffness. Future studies with larger, more diverse populations and a control group should incorporate multivariable regression analyses to more accurately account for these potential confounders. It is important to note that the HRV method is not without its limitations. Indeed, reference values for some of the analyzed parameters have yet to be definitively established for various clinical populations. As such, their interpretation is based on previous analyses from other studies. It should be noted that the study in question did not directly measure arterial stiffness, AT was used to make indirect measurements. Further studies are needed to elucidate the exact pathophysiological mechanisms of autonomic nervous system dysfunction and cardiovascular hemodynamics in PR patients and their impact on the development of cardiovascular complications.

## 5 Conclusions

In patients with newly diagnosed PR, an association was identified between sympathetic-parasympathetic balance, as measured by HRV, and the hemodynamic profile, as assessed by ICG and AT. The findings indicated an association between lower indices of acceleration, velocity, and the Heather index, which are indicative of dysfunction in the mechanical efficiency of the heart in pumping blood through the circulatory system, and a shift away from parasympathetic influence. Conversely, heightened augmentation index and augmentation pressure, signifying diminished blood vessel elasticity, were also correlated with autonomic nervous system imbalance, manifesting as a shift away from parasympathetic influence. It is important to note that the cross-sectional nature of the study does not permit the clear determination of cause-and-effect relationships, nor the explanation of the mechanisms of interdependence between the autonomic nervous system, the cardiovascular system and prolactinoma. However, the individualized assessment of PR patients by ICG and AT and HRV may prove useful in identifying cardiovascular dysfunction at an early stage and facilitating the implementation of appropriate therapeutic decisions. The results obtained from this study encourage further research to be conducted into their clinical and prognostic significance.

## Data Availability

The original contributions presented in the study are included in the article/supplementary material. Further inquiries can be directed to the corresponding author.
